# Is It High Time to Increase Elite Soccer Substitutions Permanently?

**DOI:** 10.3390/ijerph17197008

**Published:** 2020-09-25

**Authors:** Gustavo R. Mota, Izabela Aparecida dos Santos, Rhaí André Arriel, Moacir Marocolo

**Affiliations:** 1 Exercise Science, Health and Human Performance Research Group, Department of Sport Sciences, Institute of Health Sciences, Federal University of Triangulo Mineiro, Uberaba 38025-350, Brazil; grmotta@gmail.com (G.R.M.); izabelaeduca94@hotmail.com (I.A.d.S.); 2 Exercise Physiology in Health and Human Performance Research Group, Department of Physical Education, University of Uberaba (UNIUBE), Uberaba 38055-500, Brazil; 3 Physiology and Human Performance Research Group, Department of Physiology, Federal University of Juiz de Fora, Juiz de Fora 360360-900, Brazil; rhaiarriel@bol.com.br

**Keywords:** coronavirus, football, rules, sports medicine, prophylaxis

## Abstract

Rules determine how team sport matches occur. Match-induced fatigue is specific to each sport, and may be associated with injury incidence. For example, the injury rate in soccer is distinctly higher during matches than in training sessions. Understanding the differences between team sports rules might be useful for enhancing rules (e.g., safer sport). Therefore, this study aimed to evaluate the impact of the rule-induced physical demands between soccer, futsal, basketball, and handball, focusing on substitution rules. Data from the elite team sports’ rules (e.g., absolute and relative court dimensions; the number of players, substitutions allowed, total game time, time-outs) were collected, including the changes due to the coronavirus disease (COVID-19) pandemic in soccer substitutions, and comparisons were performed. The data showed that soccer has higher rule-induced physical demands: e.g., substantially lower substitution rate, higher dimensions in absolute (eight to fifteen times), and relative (four to eight times) values. Simulations also showed that soccer has extremely large differences, even considering COVID-19 substitution changes (from three to up to five). We conclude that elite soccer has remarkably higher overall rule-induced physical demands than elite futsal, basketball and handball, and increasing soccer substitutions permanently (e.g., unlimited) might mitigate overall soccer demands.

## 1. Introduction

The overall load of soccer matches (i.e., cognitive decision making, tactical and technical proficiency inside a set of well-advanced physical capacities) results in postmatch fatigue which is associated with high match-induced muscle damage and inflammatory responses, dehydration, and glycogen depletion [1,2,3]. A systematic review concluded that a period of ~3 days postmatch, for example, is insufficient to fully recover homeostatic balance caused by a soccer match load [1]. Compared to basketball, volleyball, and handball, soccer is the most demanding sport with much higher muscle damage and inflammatory markers than the other sports [4]. Indeed, another systematic review showed that soccer has the largest total running distances, including high-intensity running and sprinting in comparison with futsal, basketball, and handball [5], and increments in soccer demands have been recognized through the years [6]. Barnes et al. investigated physical and technical soccer performance across a 7-season period in the English Premier League. Their data confirmed an increment of “only” ~2% in total distance covered per match, however they reported impressive increments in distances covered in high-intensity running distance (~30%), actions (~50%), sprint distance (~35%), and the number of sprints (~85%) [6]. This high-intensity increment may be a concern because there is a strong association between high-speed running and injuries [7,8]. Additionally, due to commitments for economic and entertainment reasons, soccer has presented congested schedules (i.e., multiple games within 72–96 h), which is a relevant issue for medical staff [9,10]. For instance, data over 11 seasons (from 27 teams) exhibited that matches with short recovery (≤four days) were related to augmented muscle injury rates when compared with longer recovery periods (≥six days) [11], generating the average cost of an elite player injured of ~ €500,000 (~1 month) [12]. Soccer is also associated with long-term sequelae due to the high loading on hip and knee joints [13], early osteoarthritis and poor quality of life after retirement [14]. Additionally, soccer injuries may produce a meaningful loss of time from participation, or even early retirement [15].

To confront the issues aforementioned, several studies have investigated strategies to improve recovery, and to minimize soccer-induced muscle damage and fatigue [2]. For example, compression garments [16,17], cold water immersion [2], myofascial release [18], etc. The literature has a myriad of studies seeking to find smarter training programs or better control of the training load [8], nutritional aids [19], sleep hygiene [20], and other strategies. Surprisingly, there are no scientific studies investigating the potential issues caused by the rules of the game itself. Reasoning scientifically, the rules of any sport are the “cause”, and the “way to play” is the effect. The injury rate is noticeably higher (~10 times) during soccer matches than during training sessions [21]. Therefore, rule evaluation to manage the main causes of issues (i.e., match) is necessary, since rules should often be updated to enhance any sport for safety (e.g., shin guards), prevention (e.g., time for hydration), entertainment, cleaner (e.g., video referee), etc.

On 11 March 2020, the World Health Organization announced the coronavirus disease (COVID-19) outbreak as a pandemic, and the regular sports season worldwide was interrupted. After months of interruption (~3 or 4 months), most sports leagues have resumed the season. Due to the overlap of competition schedules caused by the COVID-19 interruption, most soccer teams will face very congested schedules on returning (e.g., games every Sunday and Wednesday), which potentially increase the risk of injuries (e.g., muscle and ligament injuries) [10,11]. Thus, The Fédération Internationale de Football Association (FIFA) has changed the substitution rule (temporarily) increasing it from three substitutions to up to five for each team (each match), aiming to minimize the impact on player welfare [22]. There is no research regarding a deep investigation on the impact of potential rule-induced physical demands in soccer (e.g., area per player and fewer substitutes forced by the law of the game), and none comparing different sports in this context. Understanding the potential differences between the rules which may impact sport-specific fatigue and eventually injury risk [10] might be useful for practical applications (e.g., updating and enhancing rules for a safer/healthier sport). Therefore, this study aimed to evaluate the impact of the rule-induced physical demands among soccer, futsal, basketball and handball, focusing on the substitution rules (including changes due to COVID-19). We hypothesized that soccer would have potentially higher rule-induced physical demands than other team sports, even considering the changes due to COVID-19.

## 2. Materials and Methods

### 2.1. Experimental Design

In order to meet the aims of this study, first, rule-specific information for the international top men (elite) was obtained from each team sport selected. Two authors independently highlighted which rules (in each sport) might have an impact on the physical demands of the players (e.g., total distance covered). For example, the size of the goal (or basket) has a minimal potential effect on physical demands. On the other hand, the dimensions of the court/field (absolute area and relative area per player), time of playing, and the number of substitutes (absolute and relative) logically impact the demands of the sport. After a consensus between the two authors, the data were collected. Then, data were organized in Excel sheets for calculations (e.g., percentage of players available—relative substitutions allowed/total players available). Quantitative and qualitative analyses were performed and confronted with the literature already existent on physiological (e.g., muscle damage and inflammatory markers) and time−motion (e.g., number of sprints, jumps, distances covered in several speed zones) sport demands.

### 2.2. Rules of the Team Sports

Four invasive team sports (i.e., soccer, futsal, handball, and basketball) were selected because these sports have several similarities, and are popular worldwide. All are invasive intermittent team sports, have body contact, require quick (and accurate) decision making and optimum scanning (reading the game), and the purpose is to score a goal or a basket on the opponent’s territory [23,24].

The specific rule information of each team sport was obtained from official websites in June of 2020: soccer, futsal [25], basketball [26], and handball [27]. To meet the current research aims, specific information from the rules which may impact the physical demand of the players was collected. For instance, information about the number of players on the field (soccer) or court (other team sports), availability of substitutes on the bench area and when they are allowed to play (including the substitutions changes due to COVID-19), time load of each team sport, time-outs, field/court dimensions and the relation between and among that information.

### 2.3. Number of Games per Season

The number of games per season (2018–2019) from the top four teams (international, men, elite) of each sport were obtained from websites of each team or official federation. The teams for each sport are presented as a table in the supplementary document (Table S1).

### 2.4. Data Analysis

This study developed a descriptive, cross-sectional design, therefore quantitative data presentation is essentially descriptive in nature. Due to the nature of this study (i.e., there is only one rule for each sport), the data were not judged from a traditional statistical point of view (e.g., *p* value, mean values, and standard deviation). Alternatively, a qualitative analysis was performed, conducted by two authors focusing on the potential practical implications. All other authors read this analysis carefully, and edits have been combined. Such kind of data analysis (e.g., progressive statistics and case research) has been used in Sports Medicine and Sports Science fields [28,29].

## 3. Results

### 3.1. Rules of the Team Sports

Data about the number of players, substitutions, time (total, breaks, time-outs), and dimensions of the field/court are presented in Table 1. Overall, soccer rules demand higher dimensions of the field and lower substitutions, both in absolute and relative values.

Soccer has no time-out during the game, but futsal, basketball, and handball do not have this rule. The offside rule may increase the physical demand (please, see discussion).

Table 2 shows simulations to equate the soccer dimension, changing the current court dimensions.

**Figure 1:**
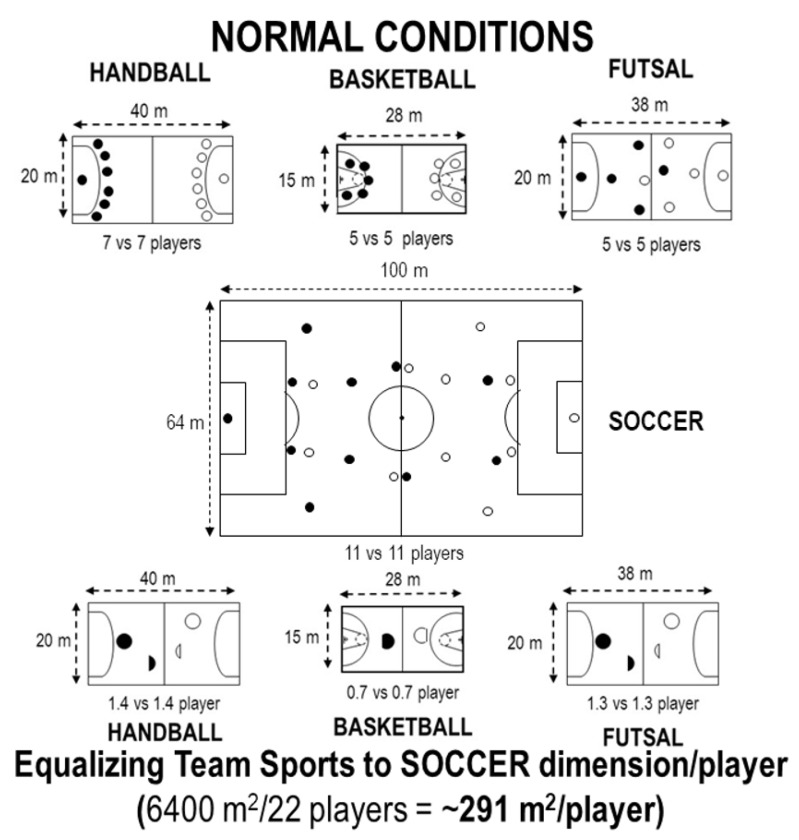
Regular dimensions and number of players on the field/court (top). At the bottom, simulations to equate soccer (~291 m^2^/player) to other team sports’ dimensions by decreasing the number of players (i.e., handball, basketball and futsal).

**Figure 2:**
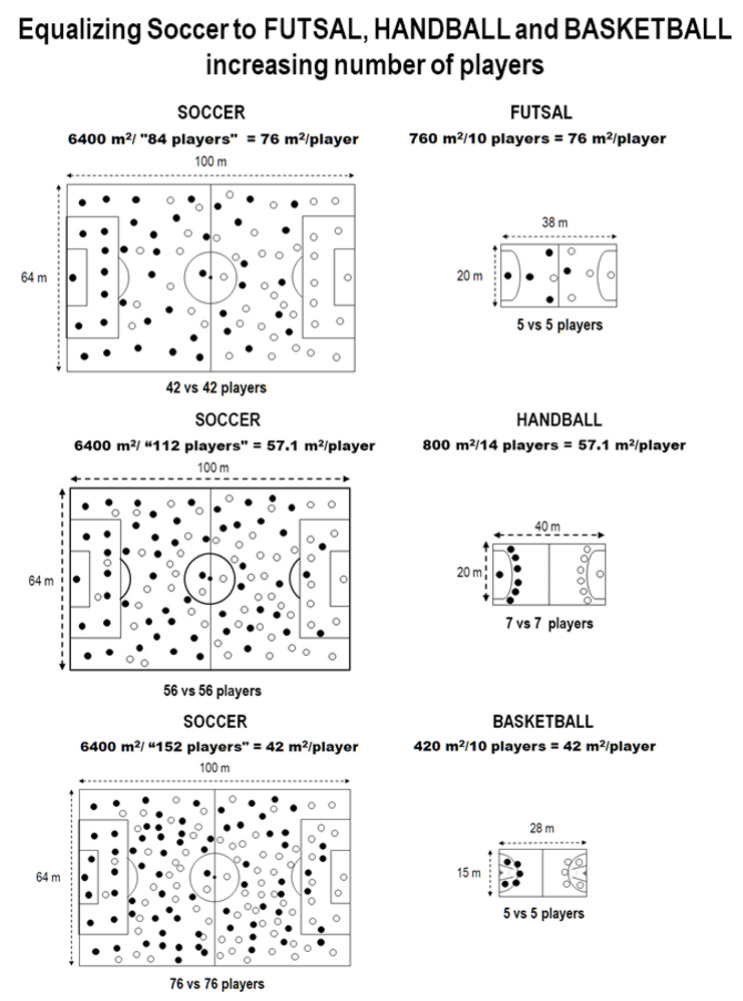
Simulations to equate soccer (i.e., to have similar m^2^/player) to other team sports’ relative dimensions, by increasing the number of soccer players.

Simulations to equate soccer to other team sports by decreasing the number of players of the other team sports or increasing the number of soccer players are shown in Figure 1 and Figure 2.

### 3.2. Number of Games per Season

Basketball presented the highest number of matches per season, followed by soccer and futsal (similar), and lately handball in top clubs (Table 3).

**Figure 3:**
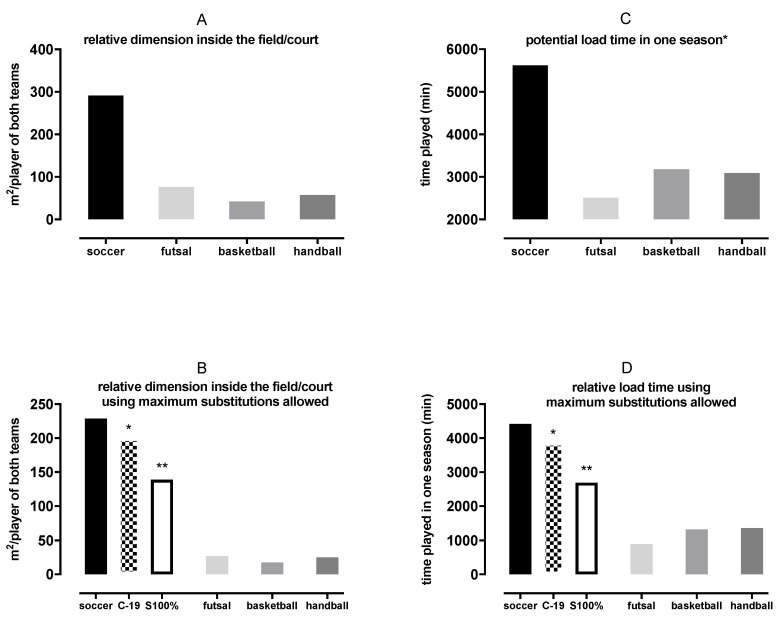
Proportion between dimensions of the fields/courts (**A**,**B**), considering two opposing teams (e.g., soccer 11 vs. 11 = 22 total). Panel (**A**) shows values related to the regular number of players on the field/court. Panel (**B**) shows the values using the maximal players involved in a game according to the regular rule for substitutes (see E in Table 1); * C-19 means the increased maximum substitutions allowed for soccer (from 3 to 5), due to postponing the calendar caused by the COVID-19 pandemic; ** S100% represents a hypothetical simulation if soccer could make unlimited substitutions (i.e., using total players available in a game (i.e., 23 each team)). So, 6400 m^2^ (soccer area)/46 players (23 × 2 teams) = 139 m^2^/player. Panel (**C**) shows a ratio between total game time X number of matches (e.g., soccer 90 min × 62.5 matches/season [Table 3] = ~5625 min/season). Panel (**D**) presents the load time using the maximum substitutions allowed in each sport. Note that for soccer we added the C-19 * and S100% ** simulations to equate (like panel B explanation).

The proportion between dimensions of the field/courts, number of players, the ratio between total match time and number of matches/season and substitutions simulations are shown in Figure 3.

## 4. Discussion

This study shows for the first time that elite soccer presents remarkably higher overall rule-induced physical demands than futsal, basketball, and handball, and increasing elite soccer substitutions permanently (e.g., unlimited) might mitigate the overall soccer demands. Our findings corroborate our hypothesis, and also studies involving time−motion and physiological demands [4,5]. The principal reason is the higher surface area of the soccer field, in both absolute (eight to fifteen times) and relative terms (per player; four to eight times), than those of the other sports here studied. The restricted possibility to replace players during the games (i.e., only three substitutions according to the regular rule) is crucial; the other team sports (i.e., futsal, basketball and handball) can limitlessly replace players. Even considering the increased number of substitutions during the match due to the COVID-19 changes (i.e., from three to up to five substitutions), the discrepancy in the soccer rule-induced physical demands is still too big (Figure 3). The current data (see H, I, N, O, P and Q in Table 1 and Figure 3B,D) support that soccer rule-induced physical demands may cause an overload (overall demands) on the players compared with the other team sports here investigated. As the injury rate is clearly higher (~10 times) during the matches than during the training sessions [21], the rule change due to COVID-19 seems not to be enough.

Recently a study concluded that nonstarters (i.e., substitutes who played) had a lower internal and external load, considering matches and training sessions, during congested schedules [30], confirming that matches are a crucial training component (i.e., substitutes might be detrained). Allowing soccer to increase substitutions permanently across the games, would potentially be a “game changer”. This would be easier to implement in comparison to other actions; e.g., reducing the number of competitions, since soccer has a huge economic impact [31]. Because the congested schedules in soccer are a relevant concern, and it is related to accumulated fatigue and higher risk of injuries [9,10,11,32], unlimited substitutions might be an intelligent decision. Allowing soccer unlimited substitutions (e.g., only three opportunities to make substitutions to avoid disruption or allowing the turnover of players like futsal), would likely prevent the drop in the intensity of the matches, especially in the second half [1,33,34]. Evidence exists for an improvement. For example, substitutes covered a greater high-intensity-running distance [35], and midfield substitutes covered a greater overall distance and distance at high-intensity compared to other midfield team-mates who remained on the pitch for the same period of the game [36]. Additionally, Hill et al. [37] concluded that substitutes may provide physical and/or tactical impetus, corroborating a basketball study that showed better scoring after substitutions [38].

Although a comparison among different sports is limited for obvious reasons (i.e., they “really” are different sports), the disparity between the soccer load (due to its rules) and the others is enormous. It could be considered as “villain”, the current rule of substitutions (regular and COVID-19 alteration). For example, from all players available in a match (soccer: 11 playing and 12 on the bench), the soccer coach can use only 13% or 21.7% (three players according to the regular rule and five during COVID-19) against 100% in the other sports (Table 1). Besides, in all other sports here investigated, a replaced player can play again. A long time ago, a study compared the epidemiology of injuries between soccer and handball concluding that a modification in soccer rules concerning substitutions was a must [39]. The author showed that 80% of the soccer players had to wait on the field despite an injury because all the substitutions had already been done, probably worsening the injury [39]. If, in 1984, it already was nonsensical, nowadays we cannot find an adjective to mention, since soccer matches are now much more demanding [1,4,6], and the number of games per season probably also. Unlimited substitutions potentially would reduce the injury risk during a soccer match/season, which can improve team performance since an 11-year follow-up of the UEFA Champions League concluded that injuries affect team performance negatively in soccer [40]. Recently due to the COVID-19 pandemic, two more substitutions per match were allowed (up to five total) in soccer. The contradiction is that the reason is to “protect” the physical integrity of the players [22]. Why not release the substitutions regularly, if the reason is to prevent injuries? In the same way that coaches change the rules during small-sided games (e.g., different number of players, smaller area per player) during training to target specific effects [41], why not change the substitution rule in elite official matches to obtain the benefits?

Beside the substitution limitations, elite soccer is the only sport (here investigated) which does not allow the substituted player to return to the same match, has no time-out (i.e., no chance for brief recovery), and has the offside rule (which obligates the players to move back and forth). Such conditions require even more physical effort from elite soccer players. In this sense, it is interesting to note that the time load would be reduced by ~40% if soccer could use all players available (Table 1, Q). In our data, we performed simulations to equate soccer to other sports by increasing the court dimensions (Table 2, Figure 1 and Figure 2). It is relevant to realize the huge changes that are necessary to have similar conditions. For instance, it does not make sense a basketball match “1 vs. 1” (Figure 1), or a soccer match with 76 vs. 76 players on the field (Figure 2).

Our data showed that basketball has the highest number of matches during a season, while soccer and futsal are similar, and handball has the lowest number of matches per season (Table 3). Although soccer has fewer matches per season than basketball, when we investigated the ratio of matches and time load exposure, soccer shows around a two-fold greater time played (matches and season) than the other sports (Figure 3C,D). Even assuming together (i.e., futsal plus basketball plus handball) the soccer relative load time is higher (Figure 3D). When the COVID-19 changes in substitutions is considered, the scenario is better, but still, soccer alone is most demanding (i.e., higher time load per season). However, when allowing unlimited substitutions in soccer (simulation), then, soccer would have lower load time per season than the other three team sports “together” (Figure 3D). Therefore, it seems crucial to increase the number of substitutions in elite soccer, beyond the COVID-19 changes. The same reasoning would apply to the dimension of each team sport (Figure 3A,B). Although the field area would not be equal, the increment on soccer substitutions would make elite soccer less physically stressful.

It has been shown that soccer promotes higher metabolic demands [1], and causes greater inflammatory responses and muscle damage, compared with handball, basketball and volleyball [4]. Lastly, but not least, beside the higher overall physical demand from elite soccer, especially on the lower limbs (to run, sprint, jump), all the technical skills are performed (e.g., passes) by the “same” lower limbs. Although the lower limbs are fully in demand (e.g., runs, sprints, accelerations, decelerations) during a basketball or handball game, they may not kick the ball. Soccer-induced fatigue worsens the quality of skills such as passing and shooting [42], which are decisive for soccer. Indeed, lower extremities are more injured in soccer [43]. Those facts are additional points to suggest unlimited substitutions during elite soccer games (permanently) as a strategy to improve recovery, prevent injuries, and improve performance.

One limitation of the current study is that it is difficult to compare different sports, since they are naturally different. We also cannot confirm that all players participate in all the matches during a season. However, the same might happen for other sports too. Thus, such limitations do not obscure our conclusions. Although the increment in substitutions might be an interesting strategy to mitigate the high overall fatigue from elite soccer, we acknowledge that many substitutions during a game may influence a team’s tactics. Therefore, the coaches should handle it to take advantage of this possibility. Another potential limitation of the current study is that we did not consider the surfaces. The playing surfaces of the field (soccer) and courts (futsal, basketball, and handball) of these sports are different and may influence the physical demands.

## 5. Conclusions

We conclude that elite soccer has remarkably higher overall rule-induced physical demands than futsal, basketball, and handball, even acknowledging the change in substitutions due to the COVID-19 pandemic. As a practical application, allowing the increase of elite soccer substitutions permanently (e.g., unlimited) across the game is a simple strategy to improve recovery and to mitigate the enormous overall soccer demands. Since soccer injury rate is much higher during matches (vs. training sessions [21]), this rule change may help to face congested schedules and benefit injury prevention.

## Figures and Tables

**Table 1:** Description of the rules and mathematical outcomes obtained of each team sport, focusing on the substitutions.

Variables		Soccer	Futsal	Basketball	Handball
players on field/court	A	11	5	5	7
available substitutes *	B	12	9	7	9
total players available	C (A + B)	23	14	12	16
substitutions allowed	D	3	Unlimited ***	Unlimited ***	Unlimited ***
maximum players involved in a game (all substitutions allowed)	E	14 (A + D)	14 (C)	12 (C)	16 (C)
COVID-19: substitutions allowed **	F	5	Unlimited ***	Unlimited ***	Unlimited ***
COVID-19: maximum players involved (all substitutions allowed)	G (A + F)	16	14	12	16
relative substitutions allowed/total players available	H (D/C)	13%	100%	100%	100%
relative COVID-19: substitutions allowed/total players available	I (F/C)	21.7%	100%	100%	100%
a substituted player can return to the game?	J	No	Yes	Yes	Yes
total game time and [half-time] (min)	K	90 [15]	40 [15]	40 [19]	60 [10]
total time load (A*K; min)	L	990	200	200	420
L/E (min/player)	M	70.7	14.3	16.7	26.3
COVID-19: L/G (min/player)	N	61.9	14.3	16.7	26.3
L/C *** if soccer could use all players available (min/player)	O	43	14.3	16.7	26.3
mitigation using COVID-19 substitutions allowed (from M to N)	P	−12.4%	−0%	−0%	−0%
mitigation using all substitutions allowed (from M to O)	Q	−39.2%	−0%	−0%	−0%

* international and official competitions—minimum dimensions (for soccer); maximum allowed by rules; ** soccer has changed number of maximum substitutions allowed from 3 to 5, due to postponing the regular calendar caused by the COVID-19 pandemic; *** unlimited, using total players available (C).

**Table 2:** Simulations to equate to soccer dimensions (6400 m^2^/22 players = ~291 m^2^/players), changing the current court dimensions.

Variables		Soccer	Futsal	Basketball	Handball
field/court dimension length x width (m) *	A	100 × 64	38 × 20	28 × 15	40 × 20
total field/court dimension (m^2^) *	B	6400	760	420	800
normalized total dimension (% of soccer)	C	100%	11.9%	6.6%	12.5%
number of players inside field or court	D	22 (11 vs. 11)	10 (5 vs. 5)	10 (5 vs. 5)	14 (7 vs. 7)
number of players on field or court (% of soccer)	E	100%	45.4%	45.4%	63.6%
area per player (B/D) (m^2^/player)	F	291	76	42	57
area per player (B/D) (% of soccer)	G	100%	26%	14.4%	19.6%
“increasing A” (m) to equalize soccer (F)	H	-	74 × 39	74 × 39	90 × 45
“increasing B” from H (m^2^)	I	-	2886	2886	4050
equalized area per player (I/D) (m^2^/player)	J	291	289	289	289

* dimensions for international matches—minimum dimensions (for soccer).

**Table 3:** Number of matches of the season 2018–2019 for all team sports evaluated (men, elite teams).

Soccer	Futsal	Basketball	Handball
Liverpool (62)	Magnus (57)	CSKA Moscow (71)	Vardar Skopje (50)
Tottenham Hotspur (62)	Sporting Lisboa (58)	Real Madrid (86)	Barcelona Lassa (53)
Flamengo (76)	Corinthians (74)	Barcelona Lassa (84)	MVM Veszprém (51)
River Plate (50)	Barcelona (62)	Anadolu Efes (77)	Vive Targi Kielce (52)
62.5 *	62.8 *	79.5 *	51.5 *

* Mean.

## Supplementary Materials

The following are available online at https://www.mdpi.com/1660-4601/17/19/7008/s1, Table S1: Top four teams to obtain the number of games per season (2018–2019), international, men, elite of each sport.
