# Supplementary material for: Is It High Time to Increase Elite Soccer Substitutions Permanently?
Source: Int J Environ Res Public Health. 2020 Sep 25;17(19):7008. doi: 10.3390/ijerph17197008 (PMC7579365; doi:10.3390/ijerph17197008)
Supplement: Supplementary file 1 [file ijerph-17-07008-s001.pdf]

Table S1. Top four teams to obtain the number of games per season (2018-2019), international, men, elite of each sport.

soccer	futsal	basketball	handball
Liverpool	Magnus	CSKA Moscow	Vardar Skopje (50)
Tottenham Hotspur	Sporting Lisboa	Real Madrid	Barcelona Lassa (53)
Flamengo	Corinthians	Barcelona Lassa	MVM Veszprém (51)
River Plate	Barcelona	Anadolu Efes	Vive Targi Kielce (52)

a) soccer - the best of Europe UEFA Champions League and South America Libertadores; b) futsal – the top four on the last World Intercontinental Futsal Cupfutsal. The information from Boca Juniors (from Argentina) was not found, so data from Sporting Lisboa (2018-2019 champion of the UEFA Futsal Champions League) was used; c) basketball – top teams of UEFA Basketball League; d) handball – top teams of the European Handball Federation.
